# A bibliometric analysis of mental health among high school students

**DOI:** 10.3389/fpsyt.2024.1433897

**Published:** 2024-11-25

**Authors:** Shilong Song, Wenbing Yu, Shuoqi Li, Wenze Sun, Jiannan Fu, Qi Cheng

**Affiliations:** ^1^ Teaching Center of Fundamental Courses, Ocean University of China, Qingdao, Shandong, China; ^2^ Institute of Sports Science, Nantong University, Nantong, Jiangsu, China

**Keywords:** high school students, mental health, visual analysis, research hotspots, frontier analysis

## Abstract

**Background:**

In recent years, with the increase in academic pressure and changes in social environment, especially after the outbreak of COVID-19, there has been a significant impact on the mental health of high school students. This trend, which is concerning, requires a systematic bibliometric analysis to develop effective preventive and remedial measures.

**Objective:**

This study aims to identify and analysis the research hotspots, frontiers and emerging trends in the field of high school students’ mental health over the past two decades using CiteSpace software. These findings provide important insights that can shape future research agendas and guide targeted interventions to improve the mental health outcomes of this vulnerable group.

**Methods:**

This study utilized the Web of Science Core Collection database as its data source to retrieve literature pertaining to high school students’ mental health from January 1, 2004, to January 1, 2023. The initial search yielded 1,764 relevant documents. After manually screening to exclude duplicates, conference proceedings, announcements, and irrelevant documents, a total of 1,748 relevant documents were retained. The research employed the CiteSpace 6.2.R4 tool to evaluate various bibliometric indicators of the included literature, including statistics on institutional and author publication volumes, as well as co-citation analysis.

**Results:**

A total of 1,748 English-language documents were retrieved, showing an upward trend in publications on high school students’ mental health from 2004 to 2024. The research hotspots primarily focus on COVID-19 pandemic, depression, bullying, substance abuse, sexual behavior, Physical Education and their relationships with the mental health of high school students. Future research trends may focus on studies investigating the relationships between the use of electronic cigarettes, sleep disorders, internet addiction, and the mental health of high school students.

**Conclusion:**

In addition to the six major research hotspots of COVID-19 pandemic, Depression and stress, Suicidal ideation, Bullying, Sexual behavior and Physical education. Researches should pay more attention to the use of electronic cigarettes, sleep disorders, and Internet addiction among high school students.

## 1 Introduction

High school students are at a critical stage of growth and development, making them particularly vulnerable to psychological health issues influenced by puberty, interpersonal interactions, and social environments. According to the World Health Organization, over 13% of adolescents worldwide experience psychological health issues (1), which has garnered significant attention from scholars. Research indicates that these issues have become increasingly severe in recent years. For instance, a nationwide representative study in Sweden revealed a rising trend in adolescent psychological problems from 2002 to 2018 (2). Similarly, a national study in Australia reported that the prevalence of mental disorders among high school students reached 39.6% in 2020-2021, marking a 50% increase from the 2006 survey (3). It is evident that psychological health issues among high school students are serious. These problems not only jeopardize students’ academic performance and daily lives but may also lead to suicidal impulses. A survey conducted by Iyanda et al. (4) involving 3,632 high school students found that the 12-month prevalence rates of suicidal ideation and plans exceeded 50%, with suicide currently being the second leading cause of death among adolescents globally (5). Consequently, the psychological health issues faced by high school students have attracted considerable scholarly attention, making it essential to explore the research hotspots and emerging trends in this field to promote research and development aimed at alleviating these issues.

Cite Space is a scientific citation visualization analysis software that can display the trends and movements of a discipline or knowledge field over a certain period, forming an evolutionary process of several research frontiers. To date, no scholars have employed bibliometric methods to conduct a quantitative analysis in the field of high school students’ mental health. In this study, Cite Space 6.2.R4 software was used to perform a visual analysis of the literature in the field of high school students’ mental health from the Web of Science Core Collection database, aiming to identify the research hotspots frontiers and emerging trends, thereby providing references for future researchers.

## 2 Materials and methods

### 2.1 Literature sources and retrieval strategies

**Figure 1:**
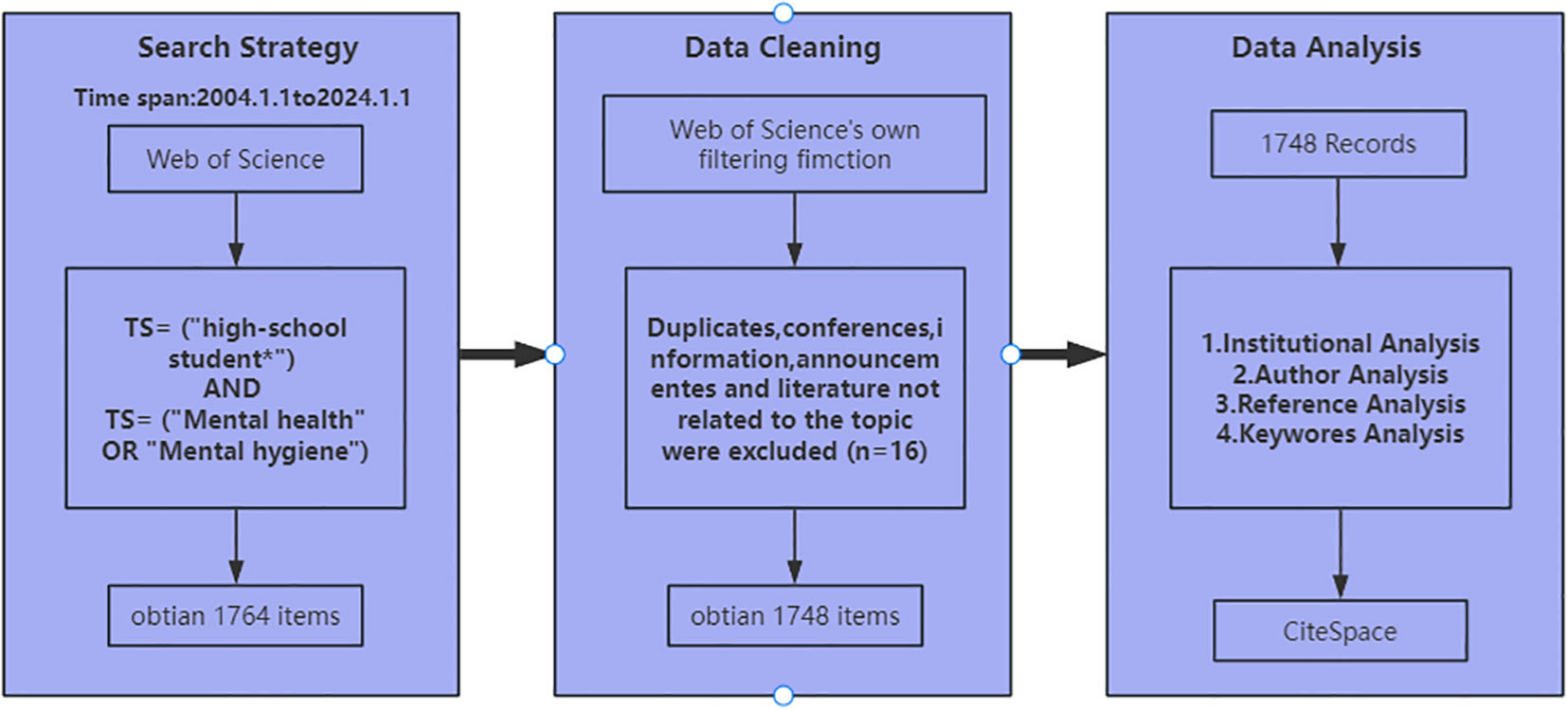
Literature screening process.

The present study utilized the Web of Science Core Collection database as the source of data. The time span was set from January 1, 2004 to January 1, 2024. The English search string was: TS= (“high-school student*”) AND TS= (“Mental health” OR “Mental hygiene”). The retrieval date ranged from January 1, 2004 to January 1, 2024. To ensure the quality and credibility of the literature research, a manual screening was conducted to exclude articles without authors. Consequently, 1748 valid English –language publications were obtained. The literature screening process is shown in **Figure 1**.

### 2.2 Research method

#### 2.2.1 Data extraction

Two researchers conducted the literature search in both Chinese and English using a unified search strategy. Synonymous keywords were consolidated, for example, ‘high school students’ and ‘high school pupils’ were merged into‘ high school students’, ‘anxiety’ and ‘anxiety disorder’ were merged into ‘anxiety’, etc. Disputed keywords were determined after consultation with a third party. The literature retrieved was screened in batches according to the inclusion criteria, and those meeting the conditions were included. In case of disagreement, the matter was resolved after consultation with a third researcher. Additionally, the authors’ institutions were consolidated; secondary departments or affiliated departments were subsumed under the primary institution of the authors, for instance, Southwest University and its Psychological Health Education Research Center were merged into Southwest University. The inclusion of authors was not ranked, and all were considered as a reference for the volume of publications.

#### 2.2.2 Data organization and analysis

The literature retrieved from the Web of Science Core Collection database was exported in “plain text file” format, with the record content selected as “full records and cited references.” This data was then processed using CiteSpace 6.2.R4 visualization analysis software.

#### 2.2.3 Parameter settings

The time span for the literature sources was set from January 2004 to January 2024. The number of retrieved literature was 1748. When setting the Years Per Slice, in order to observe research trends over a longer time span, reduce the interference of short-term fluctuations on the study, and prevent data fragmentation caused by too short a time setting, the Years Per Slice was set at two-year intervals. The metric thresholds (Top N per slice, g-index) and other parameters were chosen according to the different types of nodes. Different node types were selected with varying parameter settings to conduct co-occurrence analysis, clustering analysis, and keyword emergence analysis of the included literature data, focusing on high-yield institutions, high-yield authors, and cited references. Additionally, manual integration and analysis were performed, resulting in the creation of a visual map or chart.

#### 2.2.4 Visual analysis method

This research primarily utilized CiteSpace 6.2.R4 visualization analysis software and Excel to conduct a bibliometric analysis of the retrieved literature. The visualization analysis generated various knowledge maps, focusing on word frequency, clustering, and citation analysis across aspects such as institutions, authors, and keywords. The analysis identified the leading institutions and authors in this field over the past two decades, exploring the research hotspots and emerging trends of on high school students’ mental health during this period.

## 3 Result

### 3.1 Overall characteristics of publications

**Figure 2:**
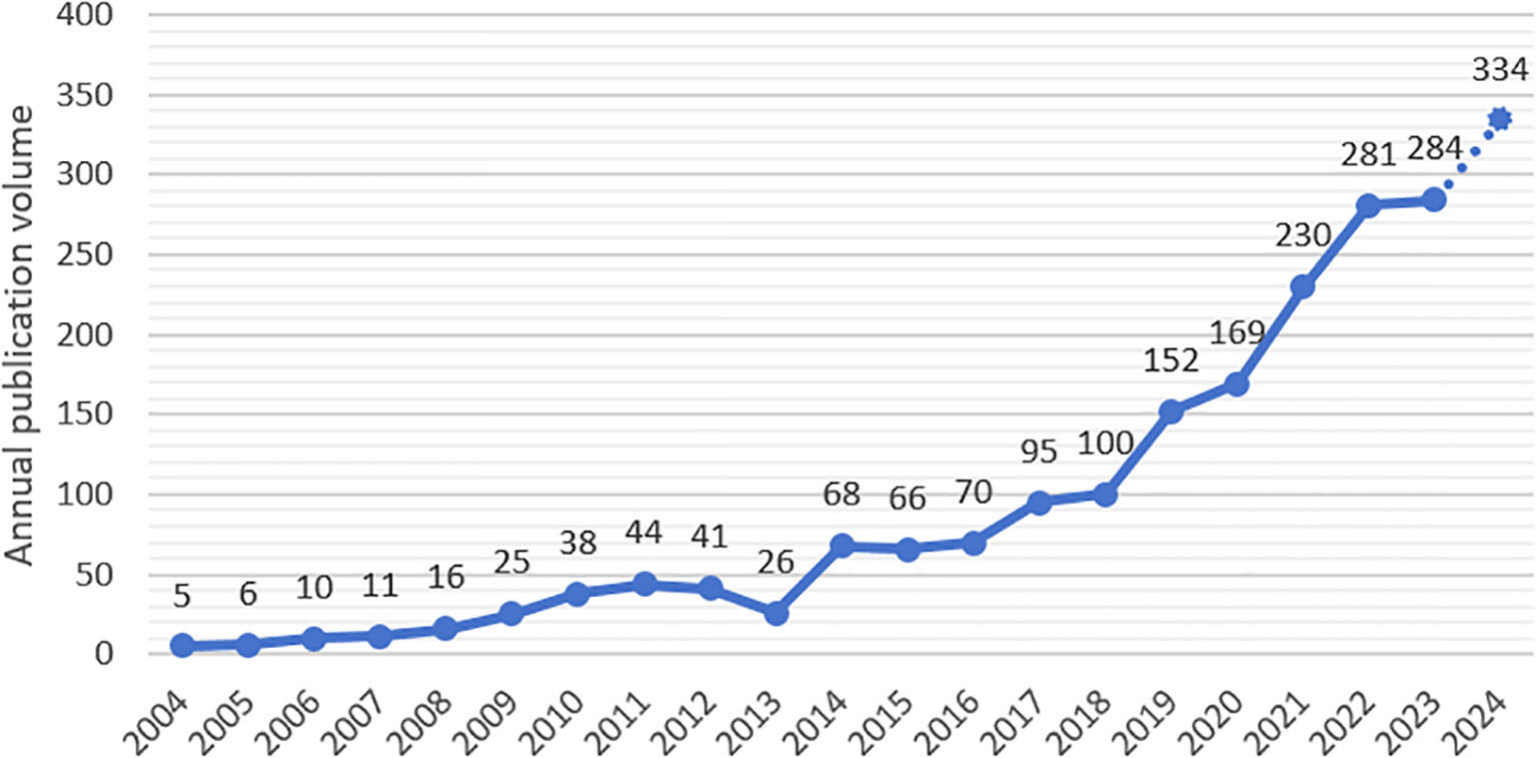
Annual publication trend of research on mental health among high school students from 2004 to 2024.

Academic journals and papers play a crucial role in documenting the outcomes of scientific research. Conducting a historical and thorough statistical analysis of literature distribution within a particular field is vital for assessing its current phase, as well as forecasting future trends and movements. The annual output of publications, derived from a statistical examination of the research data, is depicted in **Figure 2**.

The analysis reveals a general upward trajectory in the annual publication of English-language literature. From 2004 to 2012, the volume of published papers was modest, with minimal growth. A noticeable decline occurred in 2013, followed by a marked increase post-2013, particularly between 2020 and 2022. This pattern suggests an escalating severity in high school students’ mental health issues beginning in 2013, with the most significant surge anticipated during 2020-2022. It is postulated that the advent of the novel coronavirus pneumonia outbreak has exacerbated these mental health challenges, thereby catalyzing a rise in related research. Projections for 2023 to 2024 are depicted with dashed lines to denote that the publication figures for 2024 are predictive, based on the average growth rate observed over the preceding five years.

The analysis of annual publication trends indicates a significant increase in research on the mental health of high school students. Moreover, future studies in this area are expected to sustain a high level of interest. This trend also indirectly reflects the escalating severity of mental health issues, highlighting the need to explore key research hotspots and emerging trends in this field. Such insights can enhance problem identification and guide the implementation of effective intervention measures.

### 3.2 Analysis of institution

**Figure 3:**
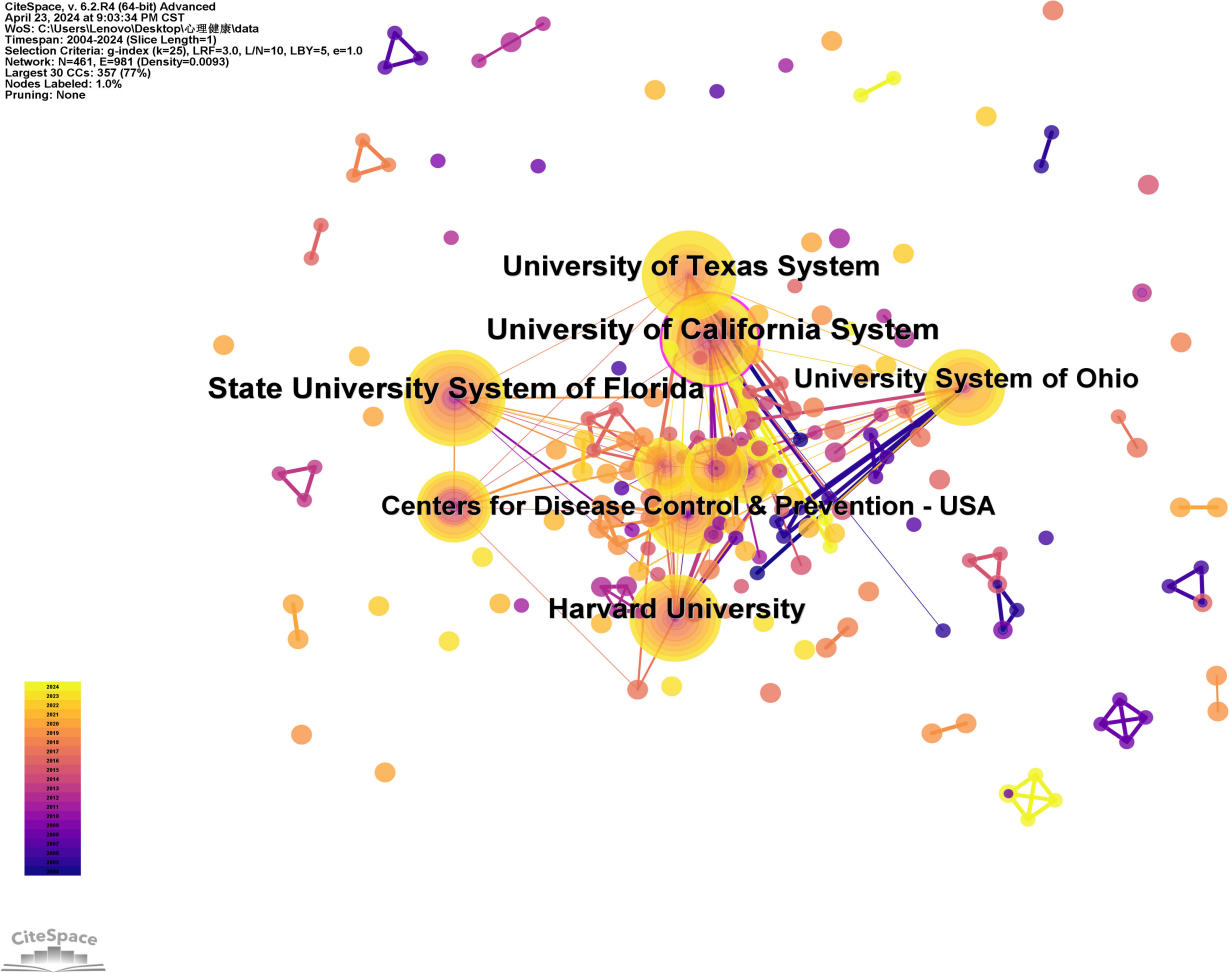
High yield mechanism diagram.

Using institutions as nodes, this study analyzes literature data sourced from the Web of Science Core Collection database, resulting in the creation of diagrams and tables for high-output institutions as shown in **Figure 3** and **Table 1**. Through the graphical and tabular data of these high-output institutions, one can observe the publication volume and influence of relevant institutions within this research field. This facilitates a more comprehensive understanding of the current research development status in this area.

The top five institutions are listed in the table below:

**Table 1** illustrates that the University of California has the highest publication volume and centrality(“Centrality” measures a node’s position and connectivity within a network, reflecting its influence and significance). This suggests the university’s exceptional productivity and authority in this field. Florida State University ranks second in publication volume, although its centrality is lower. Harvard University and the University of Texas are tied with 43 publications each, and their centrality scores are nearly identical. The ranking of institutional publication volumes indicates that universities focusing on high school students’ mental health issues are prominent. Researchers from various disciplines are active within these institutions, and this study may provide valuable references for future research directions in this area.

**Table 1:** Top 5 institutions with the most frequent publications on mental health research of high school students.

Number of Publications	Centrality	Affiliations
54	0.17	University of California
52	0.05	Florida State University
43	0.07	Harvard University
43	0.08	University of Texas
34	0.05	Ohio university

### 3.3 Analysis of authors

**Figure 4:**
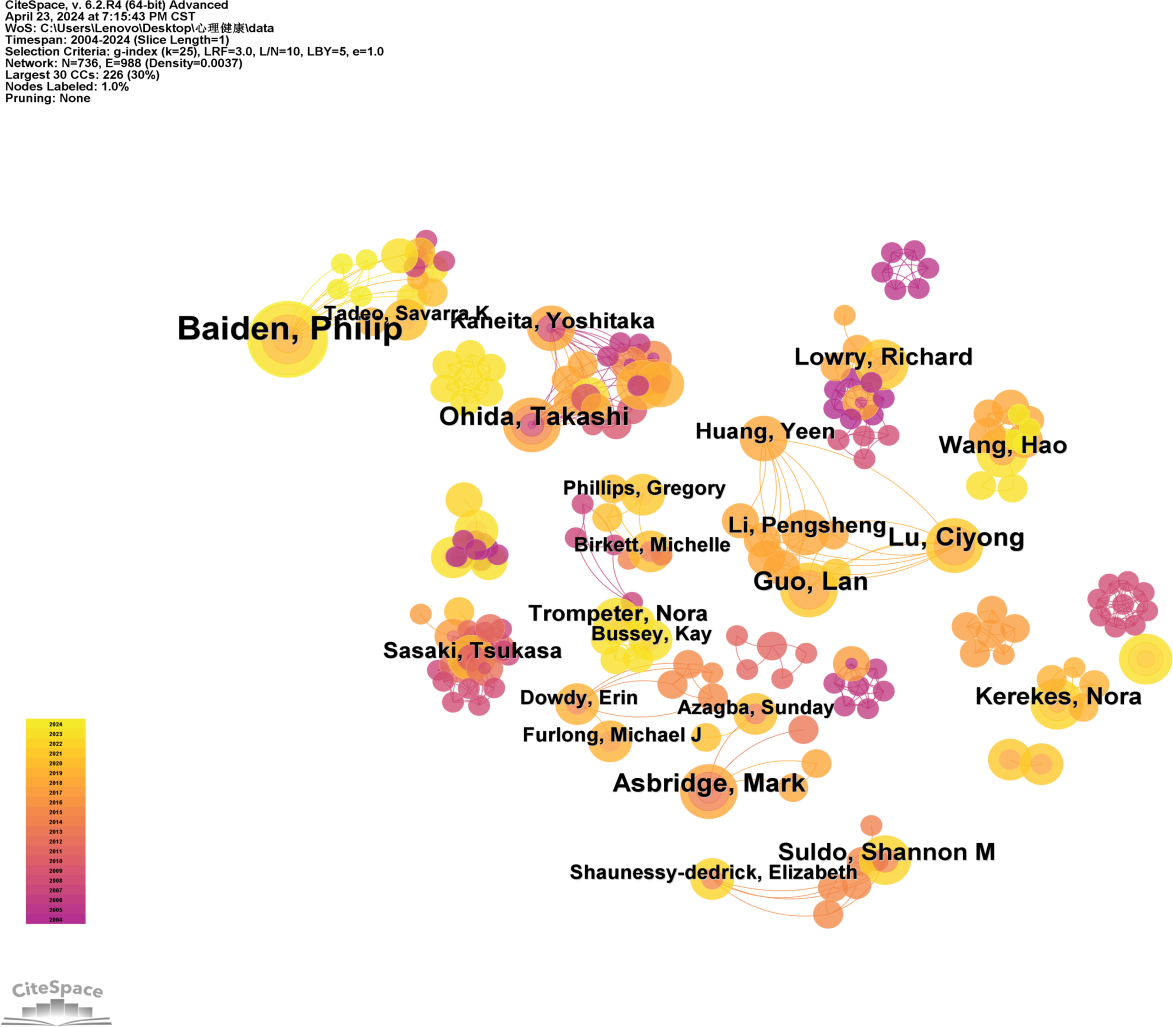
Author relationship diagram.

Using authors as nodes, this study analyzes bibliographic data sourced from the Web of Science Core Collection database and constructs diagrams and tables for prolific authors as shown in **Figure 4** and **Table 2**. This allows for the visualization of publication volume, influence, and collaboration networks of relevant authors within the research field. Such representations facilitate a more comprehensive understanding of the current research development status in this area.

The top five authors in terms of publication volume are listed in the table below:

**Table 2** reveals that the primary contributors to research on high school students’ mental health include Baiden, Philip; Leatherdale, Scott T; GuoLan; Asbridge, Mark; and Ohida, Takashi, among others. Notably, Baiden and Philip have the highest publication count with 14 papers, while Asbridge and Mark are pioneers in this area of study. Dr. Baiden Philip specializes in investigating adolescent suicidal behavior and its links to environmental factors like neighborhood violence and substance misuse. Leatherdale and Scott T. have extensively explored the effects of COVID-19 on youth. Meanwhile, GuoLan from China have focused on how coping styles mediate various mental health issues in high school students. The data indicates a lack of significant collaboration among these researchers.

**Table 2:** Top 5 authors in terms of publication frequency of research on mental health of high school students.

Authors	Number of Publications
Baiden, Philip	14
Leatherdale, Scott T	8
Guo, Lan	7
Asbridge, Mark	7
Ohida, Takashi	7

### 3.4 Analysis of co-citation and keyword clustering

Co-citation analysis of literature, which identifies the relationships in which two or more documents are cited together by one or more other documents, facilitates the identification of significant works and research hotspots within a field. This method reveals clusters of highly cited documents, providing a concise summary of thematic research hotspots in the area of high school students’ mental health and enhancing understanding of key issues. Clustering analysis of keywords, a text mining technique, aims to group keywords from a set of documents based on their semantic similarity or co-occurrence, thereby uncovering potential associations. By analyzing the co-occurrence frequency and centrality of specific keywords within a domain, researchers can identify the research hotspots these keywords represent. However, relying solely on co-citation analysis may not fully capture research hotspots due to the lower citation frequency of newly published papers. Therefore, a comprehensive analysis that integrates co-citation and keyword clustering, along with manual summarization, provides a more thorough understanding of research hotspots in the field.

**Figure 5:**
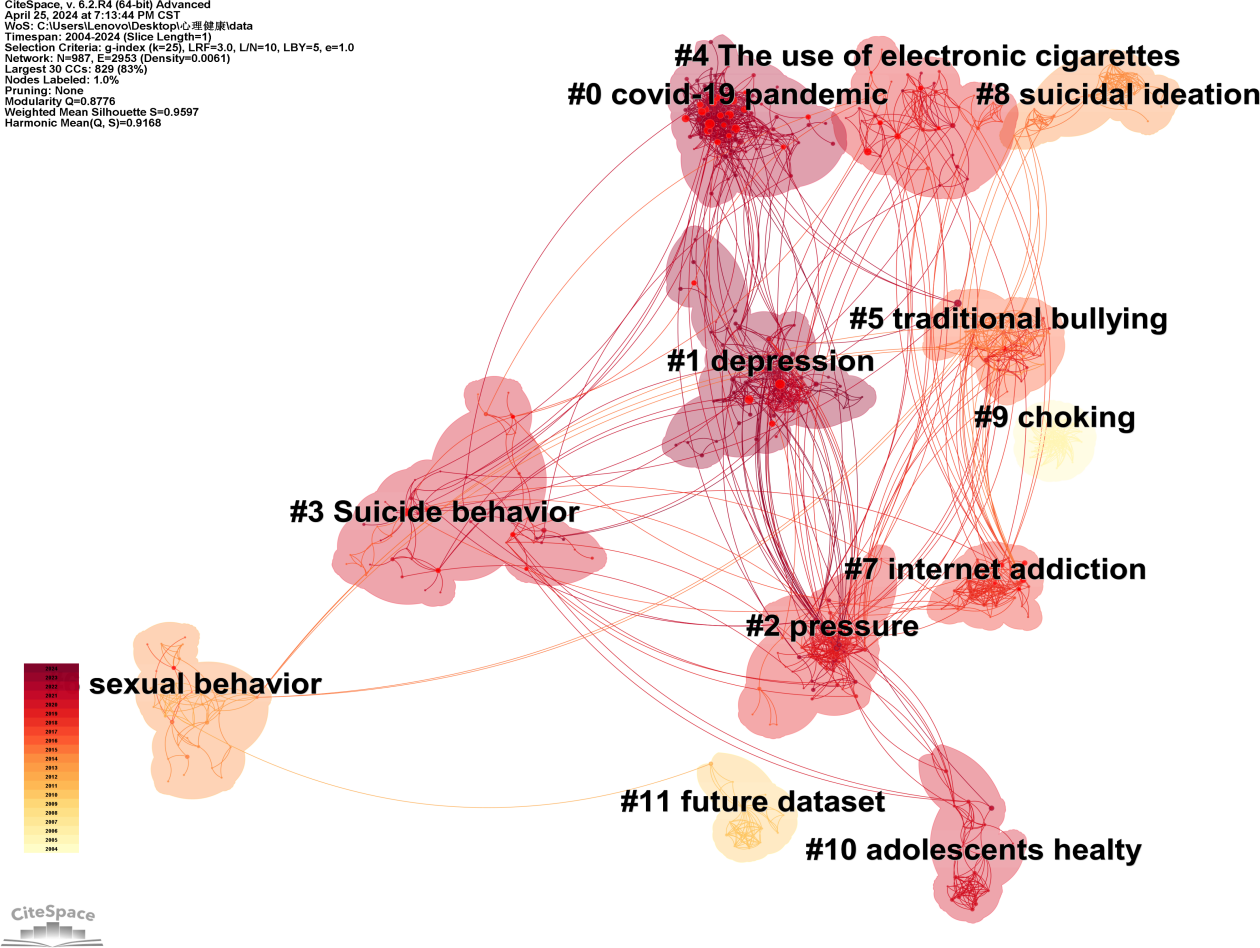
Co-citation map of mental health research literature of high school students.

**Figure 6:**
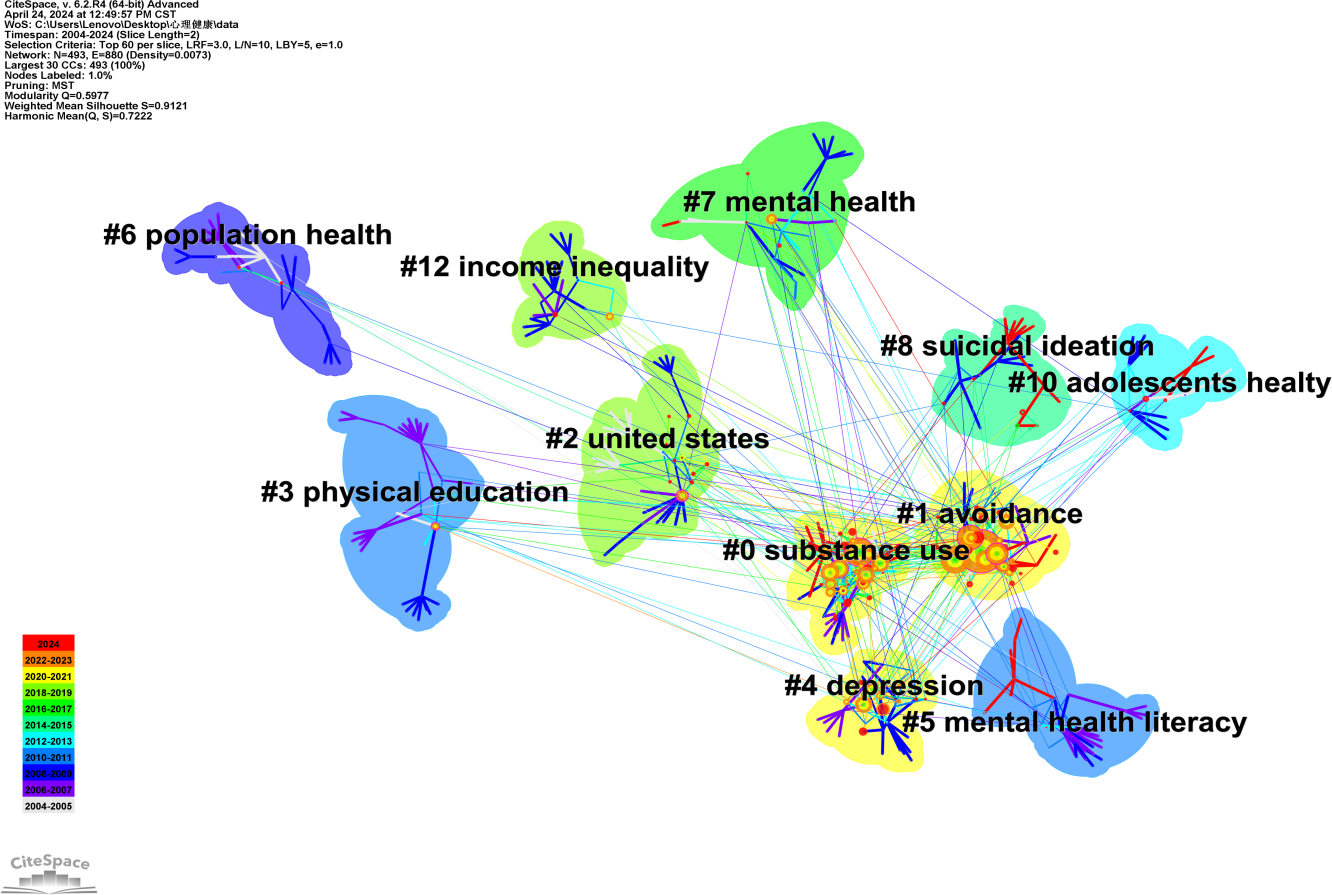
Keyword graph of psychological health research for high school students.

From the data in the figures, it is observed that the Q value for Map 5 is 0.8776 and the S value is 0.9597, while for Map 6, the Q value is 0.5977 and the S value is 0.9121, indicating that both maps exhibit significant clustering structures with high reliability. Based on the data presented in the figures, it is evident that the Q value for Graph 5 is 0.8776, and the S value is 0.9597. For Graph 6, the Q value is 0.5977, and the S value is 0.9121. When utilizing CiteSpace for bibliometric analysis, the Q and S values can assist researchers in assessing the structure and clustering rationality of knowledge maps (6), thereby enhancing the understanding of the knowledge structure and development trends within a research field. The Q value ranges from 0 to 1, with higher values indicating that the nodes in the network tend to form tighter communities or modules. An S value close to 1 suggests that the clustering results are excellent, indicating that the data points within each cluster are very cohesive and distinct from those in other clusters. Therefore, it can be concluded that both graphs exhibit a significantly high-reliability clustering structure. The cluster labels in **Figure 5** are named as follows: #0 covid-19 pandemic, #1 depression, #2 pressure, #3 suicide behavior, #4 The use of electronic cigarettes, #5 traditional bullying, #6 mental health literacy, #7 internet addiction, #8 suicidal ideation, #10 adolescents’ health. The cluster labels in **Figure 6** include: #0 substance use, #1 avoidance, #2 united states, #3 physical education, #4 depression, #5 mental health literacy, #6 population health, #7 mental health, #8 suicidal ideation, #10 adolescents’ health, #12 income inequality. By integrating two figures, we manually summarized the literature within the clusters to determine the final cluster names and exclude those irrelevant to this study. The results indicate that since 2004, the primary research hotspots in high school students’ mental health include the COVID-19 pandemic, depression and stress, suicidal ideation, bullying, sexual behavior and physical education. The discussion section will explore why these six aspects have garnered attention over the past two decades, examining new findings and recent advancements in each area to provide valuable references for other scholars.

### 3.5 Analysis of key words emergent

In the literature burst analysis, the display setting in CiteSpace is adjusted to ‘burst report’ and upon selecting ‘View’, the minimum duration for literature burst keywords is configured to one year. This setup facilitates the acquisition of data regarding the frequency and longevity of prominent keywords in the realm of high school students’ mental health research over recent years.

**Figure 7:**
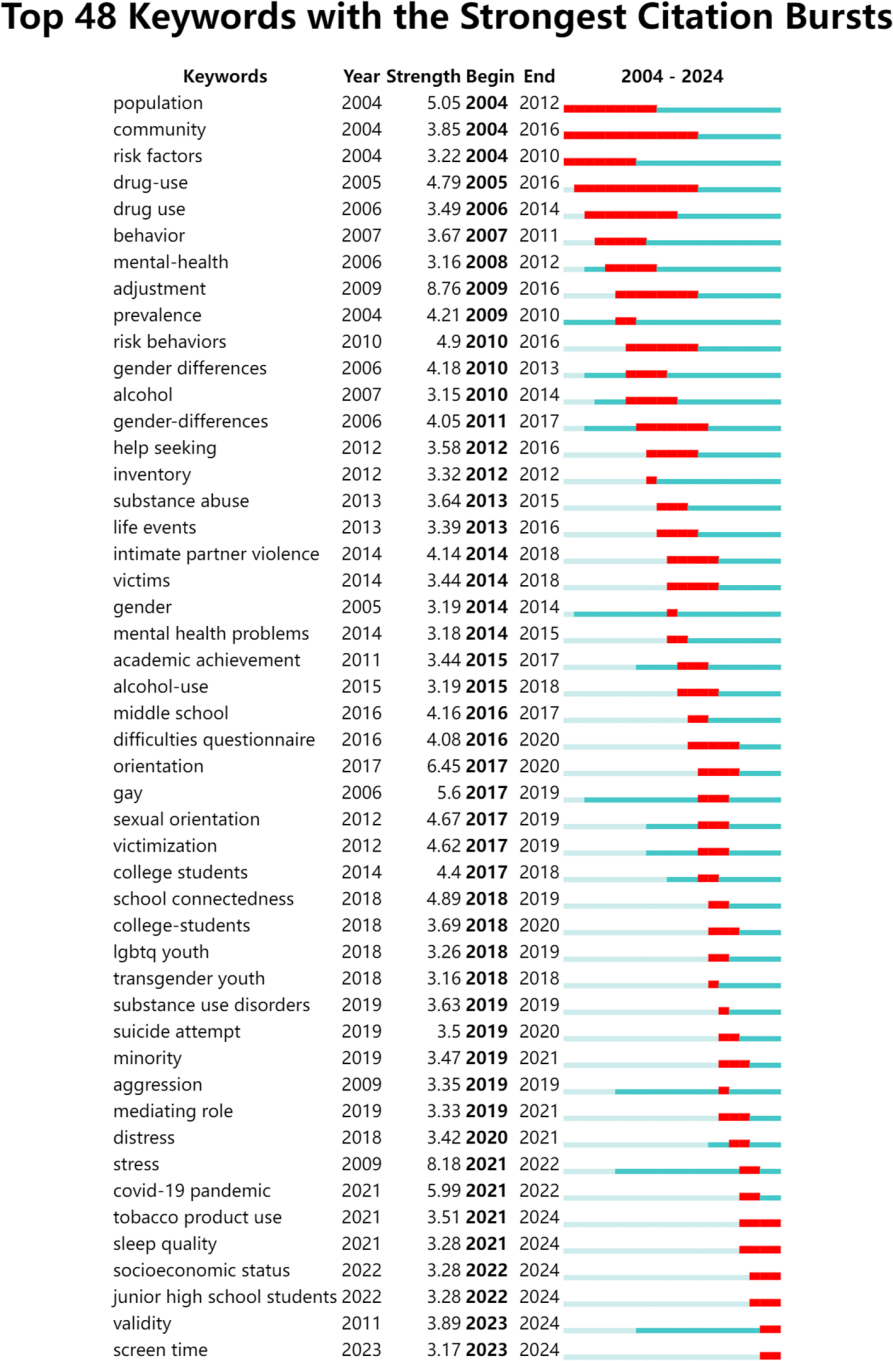
Keyword emergence map of high school students’ mental health research.

As shown in **Figure 7**. As can be seen from Keyword emergence map, there are six keywords that have experienced citation bursts (a phenomenon where a particular paper is cited significantly more frequently within a specific time period, indicating that the paper has attracted widespread attention and discussion in the academic community) continuing through January 1, 2024. After manually summarizing and excluding the irrelevant keywords “socioeconomic status,” “junior high school students,” and “validity,” we observe that future research trends in this field may focus on three areas: tobacco product use, sleep quality, and screen time. This suggests that issues related to these aspects have been prominent in the past three years and are likely to remain significant in the near future. By exploring these potential areas of focus, new discoveries may emerge that could provide substantial reference value for scholars in this field.

## 4 Discussion

### 4.1 Research hotspots and frontier analysis of hotspots

Research hotspots are areas that have garnered significant scholarly focus during a specific timeframe. These research hotspots are mirrored by the literature pertinent to that period. Unlike traditional review articles, this study utilizes CiteSpace software to present complex literature data in a graphical manner, allowing researchers to visually observe the structure, development trends, and research hotspots of the field. Moreover, traditional review articles rely on the subjective judgment and experience of authors, whereas CiteSpace software analyzes data through objective algorithms, thereby reducing subjective bias and providing more reliable and accurate results. An analysis of co-cited literature clustering distills the key directions in the study of high school students’ mental health:

#### 4.1.1 COVID-19

Cluster #0 in **Figure 5**, labeled “COVID-19 Pandemic,” represents a cluster of studies concerning the psychological issues, causes, and influencing factors among high school students during the COVID-19 pandemic. Since the outbreak of the COVID-19 pandemic, it has spread rapidly worldwide and has become a major global public health event. Windarwati and other scholars (7) have found that during the COVID-19 pandemic, the risk of mental health issues among high school adolescents increased, including psychological distress, depression, worry, anxiety, loneliness, trauma symptoms, suicide risk, sleep disorders, and socio-psychological functioning. Youngsoo Jang (8) and other researchers studied the impact of COVID-19 on high school students in South Korea, finding that the pandemic exacerbated students’ depression, anxiety, and post-traumatic stress symptoms. This likewise confirms Windarwati’s research. The causes of these issues include changes in high school students’ lifestyles due to the pandemic, such as avoiding going out, bans on gatherings, prolonged home isolation, and maintaining social distance, which can trigger depression, anxiety, and stress related to their studies, thereby endangering their mental health. However, scholar Jester, N (9) discovered that the COVID-19 pandemic also had positive effects on adolescents, as it allowed for closer and more frequent discussions with their parents during isolation. This can improve the relationship between adolescents and their parents, which to some extent may benefit the psychological health of high school students. Overall, however, the COVID-19 pandemic has had a significant detrimental impact on their overall mental health, as this crisis is the first severe crisis encountered by many adolescents in their lives, with long-term severe effects on their physical and psychological aspects, affecting their future growth and development.

The current frontier in research on the impact of the COVID-19 pandemic on the mental health of high school students concerns changes in their mental health after the pandemic. Scholars like Rong R (10) have found that while the sleep conditions of high school students worsened upon returning to campus after the lifting of pandemic restrictions, their levels of depression and anxiety steadily decreased. Moreover, transitioning from home isolation and online learning to normal school life also imposes additional stress on students.

#### 4.1.2 Depression and stress

**Figure 5**’s clusters #1 depression、#2 pressure represent and **Figure 6**’s clusters #4 depression the categorization of mental health issues among high school students, particularly depression and stress. In recent years, there has been an increasing prevalence of mental health problems among high school students, with depression and anxiety being especially prominent. A cross-sectional study by Alharbi R et al. (11) involving 1,245 high school students revealed that 34% suffered from mild depression, 35% from moderate depression, and 5.0% from severe depression. Additionally, 34.1% experienced mild anxiety, 19.5% moderate anxiety, and 9.8% severe anxiety. These data highlight the severity of depression and anxiety issues among high school students. Factors such as academic pressure, interpersonal relationships, family environment, and school bullying contribute to the development of depressive and anxious conditions during adolescence. Particularly after the outbreak of COVID-19, the shift to home isolation and online learning for high school students has been found by YanqiangTao (12) to exacerbate depression, anxiety, and academic stress. The detachment from communal learning due to home isolation, coupled with the ineffectiveness of online learning compared to classroom instruction, and prolonged social deprivation, can lead to feelings of loneliness, thereby severely impacting the mental health of high school students. From the connections among clusters in the co-citation and keyword clustering maps, it can be seen that depression and stress issues among high school students are most closely related to suicide. This indicates that problems such as depression are highly likely to lead to suicidal tendencies among high school students. Additionally, issues such as the COVID-19 pandemic, substance abuse, internet addiction, and bullying may exacerbate depression problems among high school students.

The latest frontier in this field is the investigation of the mediating role of psychological resilience in the relationship between family dynamics and depression among high school students. Ni Feirui et al. (13) conducted a cross-sectional study with a sample of 734 adolescents, finding that psychological resilience can act as a protective factor against depression, thereby modulating the relationship between family dynamics and depression. Hence, the importance of considering family dynamics, personality traits, and psychological resilience when designing effective mental health interventions for adolescents.

#### 4.1.3 Suicidal ideation

High school students are particularly vulnerable to mental health issues due to various adverse factors, with suicidal ideation being a significant concern. In **Figure 5**, clusters #3 suicide behavior and #8 suicidal ideation, as well as cluster #8 suicidal ideation in **Figure 6**, represent the clustering of high school students’ mental health and the formation of suicidal ideation. Suicidal ideation—defined as the contemplation or intent to end one’s life, whether fleeting or frequent—is a critical psychological precursor to actual suicidal acts and serves as a key indicator for potential occurrences. Variations in mental health status are the primary contributors to the emergence of suicidal thoughts, with familial and educational environments exerting a profound influence on adolescents’ psychological well-being. Research by MeiXin Zheng et al. (14) underscores the paramount impact of the father-son dynamic on high school students’ suicidal ideation, closely followed by the mother-child bond. The sense of security derived from intimate family ties provides essential emotional support, mitigating the risk of suicidal thoughts amid negative feelings. Conversely, within the school setting, bullied students exhibit compromised mental health and an increased susceptibility to suicidal ideation. Findings by Constanza Veloso B et al. (15) indicate that bullied adolescents are more prone to consider suicide, with a higher incidence of such thoughts and attempts among girls compared to boys. Furthermore, from the clustering relationships, it can be observed that suicidal ideation is more or less connected with other clusters, indicating that various mental health issues may contribute to suicidal tendencies among high school students.

At the forefront of contemporary research, Eduardo Fonseca-Pedrero et al. (16) have innovatively mapped the network structure of suicidal behavior, offering novel insights into its association with various facets of mental, emotional, and socio-emotional health.

#### 4.1.4 Bullying

**Figure 5** #5 ‘traditional bullying’ refers to the clustering of high school students’ mental health with traditional forms of bullying. Islam MI (17) et al. conducted a cross-sectional study on 2,125 adolescents and found that 25.6% of the adolescents were victims of bullying, and 12.0% were victims of cyberbullying, indicating that the issue of bullying is quite severe. High school students mainly experience traditional bullying and cyberbullying. Scholar SupaPengpid (18) and colleagues discovered that traditional bullying could lead to adverse mental health outcomes in adolescents, such as depression, anxiety, and suicide, with suicide being a particularly prominent issue. Students with symptoms of anxiety and suicidal ideation are at higher risk of involvement in school bullying, and are more likely to become bullies or victims. However, for girls, bullying others or being bullied is associated with symptoms of anxiety and suicidal ideation. Researcher Mohammad S A (19) (19) and others found that when students experience both types of bullying, the risks of drug use, self-harm, and suicide are higher than when they experience only one type of bullying. The phenomenon of bullying remains an important health issue for high school students and is associated with many mental health problems. There is an urgent need for intervention programs to assist students involved in school bullying, both in terms of their physical and mental health.

The frontier of this research field is exploring the protective role against suicidal behaviors caused by bullying. The study by Omid Dadras (20) and colleagues highlighted the importance of a supportive family environment, as well as peer and school connections, in mitigating the negative impact of bullying and cyberbullying on adolescent mental health and suicide risk. Investigating the protective role of parental, school, and peer connections against suicidal behaviors in victims is crucial.

#### 4.1.5 Sexual behavior

In **Figure 5**, cluster #6 “Sexual Behavior,” focuses on the clustering of relationships between psychological health and sexual behavior among high school students, with issues related to masturbation being most prominent within this cluster. The high school years are characterized by rapid physiological changes; however, adolescents’ cognitive development lags, remaining immature and less stable than that of adults. An influx of sexual information, combined with these developmental factors, often stimulates the adolescent brain and endocrine system, advancing the evolution of sexual psychology. Masturbation, a frequent occurrence during puberty, represents the primary sexual behavior among adolescents. Research indicates that moderate masturbation can serve as an effective means for mitigating sexual tension throughout adolescence. Conversely, improper masturbation practices can inflict considerable psychological harm, predominantly due to subsequent worries and anxiety, which constitute significant mental health challenges. Post-masturbation feelings of guilt and concern are common among adolescents, posing substantial risks to their psychological well-being. Influenced by widespread beliefs that deem masturbation detrimental, many adolescents perceive it as shameful and injurious, leading to distress for those who engage in the act. They face a dichotomy: the enjoyment derived from the act conflicts with feelings of shame regarding their behavior. This internal strife can profoundly affect their physical and mental health. Researcher Hemono, R (21) have identified a significant relationship between parental attitudes and roles and adolescent masturbation behavior. Parents who do not provide timely education on reproductive health may contribute to abnormal masturbation behaviors among adolescents. Therefore, it is essential for parents to enhance their sexual health education for adolescents, helping them prevent and manage excessive masturbation to protect their physical and mental well-being.

Current research in this area increasingly focuses on the relationship between masturbation and depression. Albobali and colleagues (22) have found that masturbation can evoke feelings of guilt, as it is often viewed negatively across various cultures and prohibited by nearly all religions. These cultural perceptions can shape sexual behavior, impact individual mental health, and exacerbate feelings of depression. In addition, clustering analysis revealed a significant relationship between high school sexual behavior, suicidal ideation, and bullying. Further research indicates that risky or irresponsible sexual behaviors are more likely to lead to suicidal ideation or attempts among high school students of both genders (23). Additionally, dating violence (24) and sexual bullying are associated with an increased risk of suicide.

#### 4.1.6 Physical activity

In **Figure 6**, cluster #3, labeled “Physical Education,” pertains to the clustering of relationships between psychological health and physical education among high school students. Practices has proved that physical exercise can exercise people’s will and character, improve mental outlook, and positively influence prevention and treatment of some psychological diseases. Through investigation and research, it is found that sports activities improve students’ psychological problems, help prevent and correct abnormal psychology, and at the same time, help them to improve their interpersonal skills and alleviate their negative emotions (25). Saengryeol et al. (26) (30) conducted two multiple logistic regression analyses to assess the impact of selected factors on suicidal ideation and stress. The findings indicate that participation in physical education can reduce the risk of suicidal ideation and stress among high school students. Consequently, physical education may serve as an effective strategy for alleviating stress in this demographic. Ortega and colleagues (27), through their research, discovered that students who engage in physical exercise for more than three hours per week outperform those who exercise less than three hours per week in terms of social, familial, physical, and emotional self-concept dimensions. This finding is highly beneficial for improving students’ mental health. Based on these results, both schools and families should place a high emphasis on the positive impact of physical education on students’ psychological well-being and encourage students to actively participate in physical activities outside of physical education classes. By observing the connections between clusters, this study identified a correlation between physical activity and both depression and substance abuse. It was noted that research on the relationship between physical activity and substance abuse (such as alcohol and marijuana) among high school students has been relatively scarce in recent years. Therefore, this area warrants further exploration in future studies.

The latest frontier exploration of this research hotspot analyzes the correlation between physical activity, self-efficacy, stress self-management, and mental health. Zhang Ge et al. (28) collected online survey data from 400 Chinese middle school students and analyzed the data using SPSS 27.0 and PROCESS 4.1. The findings indicate a positive and significant relationship between physical activities, self-efficacy, stress self-management, and mental health. Physical activities can influence mental health through the autonomous mediation of self-efficacy and stress self-management, as well as the mutual mediation between the two.

### 4.2 Emerging trends analysis

An burst analysis of keyword can explore the evolutionary trajectory of hotspots in the field and predict future development trends. This study has conducted an burst analysis of keywords, summarizing the future trends of high school students’ psychological health as follows:

#### 4.2.1 Tobacco product use

Using keyword burst analysis, keywords that experienced a sudden surge in citations within a specific time period were identified. It was found that “ tobacco product use “ experienced a burst starting in 2021 and has continued to the present. It is predicted that this topic will remain a significant research trends in high school students’ mental health in the future. The prevalence of tobacco product usage has escalated globally among adolescents in recent years, posing risks not only to their physical health but also exacerbating mental health issues. High school students, at a pivotal stage of emulation and character formation, are subtly influenced by their peers’ tobacco product usage, which can shape their own smoking behaviors. Consequently, the number of adolescent tobacco product users is on the rise. High school students are the main group using electronic cigarettes. Research conducted by Jennifer A (29) and colleagues has substantiated that tobacco product usage among adolescents is linked to a spectrum of mental health challenges, including depression, suicidal tendencies, and anxiety. These issues are magnified when adolescents concurrently use both cigarettes and e-cigarettes. This finding aligns with the research of Timothy D (30). Additionally, it is uncommon for adolescents to engage with only a single addictive substance. Thepthien et al. (31) discovered through a survey that the majority of e-cigarette-using adolescents in the United States also consume marijuana, alcohol, cigars, and other addictive substances. Jacobs et al. (32) corroborated these findings upon analyzing data from the 2017 Adolescent Behavioral Risk Survey, revealing that among 12,578 U.S. high school students, 5.2% used e-cigarettes, 9.9% used marijuana, and 7.8% were dual users, further compounding the potential harm to youth. Lucinda Lau et al. (33) used systematic review and meta-analysis to evaluate the potential association between e-cigarette use and subsequent use of psychoactive substances among young people aged 10-24. The results indicated that e-cigarette use in adolescents is a significant risk factor for subsequent use of alcohol, marijuana, and cigarettes, and may increase the risk of concurrent and simultaneous use of multiple substances. The current trend of e-cigarette usage among teenagers warrants increased scrutiny. Adolescents represent a critical target group for tobacco control initiatives. It is essential to enhance parental and peer education, with parents reinforcing behavioral restrictions, fostering self-discipline, and modeling exemplary behavior for students.

#### 4.2.2 Sleep disorders

Recent research has spotlighted sleep disorders as a burgeoning focus within the realm of adolescent mental health. A study investigating parasomnia among adolescents reported prevalence rates of arousal disorder, nightmare disorder, and sleep paralysis at 15.1%, 27.8%, and 6.8%, respectively (34). Beyond impacting academic performance, sleep disturbances have profound implications for students’ mental well-being. Wang Wanxin et al. (35) delved into the longitudinal dynamics between sleep patterns and mental health, revealing that inadequate sleep duration and suboptimal sleep quality have detrimental effects on adolescents’ mental health, precipitating symptoms of depression and anxiety. Zhang Shichen’s (36) research corroborates these findings. A spectrum of mental health issues, including depression, anxiety, and suicidal ideation, has been associated with sleep disorders in adolescents. Huang Ye et al. (37) identified a significant inverse correlation between sleep duration and symptoms of anxiety and depression, noting that individuals who sleep late are more susceptible to these conditions. It is advisable for high school students to moderate their after-school study hours and prioritize sleep, as this not only aids in diminishing anxiety and depression but also ensures adequate rest. To mitigate symptoms of anxiety and depression, maintaining sufficient sleep and allowing for necessary breaks during the day are essential.

#### 4.2.3 Internet addiction

Using CiteSpace software for keyword burst detection, it was found that “screen time” has experienced a burst starting in 2023 and continuing to the present. It is predicted that this topic may become a new research trend in the future, primarily reflecting issues related to internet addiction among high school students. With the rapid development of internet technology in recent years, the internet has permeated various fields. Its application in modern education is particularly widespread. While the internet provides convenience and speed for students, it also brings negative impacts, such as internet addiction caused by excessive use, which has severely endangered the psychological health of high school students. The mental health level of internet-addicted high school students is significantly lower than that of their peers who use the internet normally. The causes of internet addiction may include the desire to escape unpleasant feelings and fulfill certain needs. It is precisely the avoidance of depression, anxiety, and helplessness, and seeking solace online that brings a sense of satisfaction and leads to impulsive online behavior, resulting in symptoms of internet addiction. This, in turn, exacerbates symptoms of depression and anxiety, creating a vicious cycle. The oppressive feeling brought by the internet can impose psychological burdens and stress on addicted students, causing severe psychological harm and even pathological mental states.

Researchers such as Lebni, J Y (38) have found through surveys and data analysis that excessive internet use among students can lead to depression, anxiety, a decline in mental health status, and an impact on academic performance. The study by Tomaszek K (39) confirmed this and further explored the differences in the relationship between internet addiction and mental health issues among adolescents based on gender and grade level. They found that the rate of internet addiction among boys is higher than among girls, and middle school students have the highest rate of internet addiction. In high school students, the impact of internet addiction on stress and depression is more pronounced. Ye Xiaoli (40) and others using meta-analysis to investigate the relationship between depression and internet addiction. The findings indicate that adolescents with depression are at a higher risk of developing internet addiction. Moreover, adolescents with internet addiction are at a higher risk of depression, and the influence of internet addiction on the risk of depression is more pronounced. By exploring the connections between clusters, it was found that there may be a link between internet addiction and e-cigarette use among high school students. However, research in this area is currently limited, indicating a need for further investigation in the future.

## 5 Results

The annual number of articles published in the field of high school students’ mental health is steadily increasing, reflecting a growing global concern for this issue. Among institutions, the University of California has the highest number of publications and is regarded as a leading authority in this area. Philip Baiden is the scholar with the most publications in this field. However, cooperation between institutions and scholars remains limited. Over the past twenty years, research hotspots have primarily focused on the COVID-19 pandemic, depression, stress, suicidal ideation, bullying, sexual behavior, and sports, with the issue of suicide among high school students being the most critical. Current research emphasizes the use of mediating factors to intervene in depression and bullying, as well as to regulate suicidal behavior. This study also predicts that future research may explore the relationship between e-cigarette use, sleep disorders, and internet addiction in high school students. Furthermore, the findings of this study suggest several future research directions. First, the discovery that e-cigarette use can lead to the continued use of substances such as alcohol and marijuana warrants further exploration of the physiological and psychological mechanisms involved. Second, interventions for sleep disorders should be investigated, including the use of wearable devices and other technologies to monitor adolescents’ sleep patterns and quality, along with providing personalized intervention recommendations. This study identify and analysis the research hotspots and emerging trends in the field of high school students’ mental health, providing reference for subsequent scholars’ research.

## 6 Limitations and future perspectives

This study’s literature review was confined to the Web of Science Core Collection database, which inadvertently excluded certain recent publications, thereby not encapsulating the entirety of the latest research findings. Furthermore, the co-citation analysis encountered limitations, as the citation frequency for newly published papers was low, rendering it challenging to comprehensively represent the current research hotspots. Future research should aim to broaden the data retrieval scope, employ innovative analysis techniques, and endeavor to depict the research trajectory within this field with greater precision.

## Data Availability

The raw data supporting the conclusions of this article will be made available by the authors, without undue reservation.

## References

[1] Organization WH. Mental health of adolescents (2021). Available online at: https://www.who.int/news-room/fact-sheets/detail/adolescent-mental-health (Accessed August 20, 2024).

[2] Buli BG Lehtinen-Jacks S Larm P Nilsson KW Hellstrom-Olsson C Giannotta F. Trends in psychosomatic symptoms among adolescents and the role of lifestyle factors. BMC Public Health. (2024) 24:878. doi: 10.1186/s12889-024-18327-x 38515098 PMC10958834

[3] McGorry P Mei C. Youth mental health: a rising public health challenge. Australas Psychiatry. (2023) 31:245–46. doi: 10.1177/10398562231177350 37204413

[4] Iyanda AE Krishnan B Adeusi TJ. Epidemiology of suicidal behaviors among junior and senior high school adolescents: exploring the interactions between bullying victimization, substance use, and physical inactivity. Psychiatry Res. (2022) 318:114929. doi: 10.1016/j.psychres.2022.114929 36332504

[5] Pontes N Ayres CG Pontes M. Trends in depressive symptoms and suicidality: youth risk behavior survey 2009-2017. Nurs Res. (2020) 69:176–85. doi: 10.1097/NNR.0000000000000424 32058456

[6] Chen C. Citespace ii: detecting and visualizing emerging trends and transient patterns in scientific literature. J Am Soc Inf Sci Technol. (2006) 57:359–77. doi: 10.1002/asi.20317

[7] Windarwati HD Lestari R Supianto AA Wicaksono SA Ati N Kusumawati MW. A narrative review into the impact of covid-19 pandemic on senior high school adolescent mental health. J Child Adolesc Psychiatr Nurs. (2022) 35:206–17. doi: 10.1111/jcap.12370 PMC9114999 35199403

[8] Jang Y Cho HM Mok YE Chi SH Han C Yi HS. Impact of the coronavirus disease pandemic on mental health among school students in korea during the covid-19 pandemic. Soa Chongsonyon Chongsin Uihak. (2023) 34:63–8. doi: 10.5765/jkacap.220036 PMC10080259 37035795

[9] Jester N Kang P. Covid-19 pandemic: is teenagers' health in crisis? An investigation into the effects of covid-19 on self-reported mental and physical health of teenagers in secondary education. Public Health Pract (Oxf). (2021) 2:100099. doi: 10.1016/j.puhip.2021.100099 34514447 PMC8417457

[10] Rong R Xu Q Jordan KP Chen Y. Perceived epidemic impacts and mental symptom trajectories in adolescents back to school after covid-19 restriction: a longitudinal latent class analysis. J Adolesc Health. (2024) 74:487–95. doi: 10.1016/j.jadohealth.2023.09.011 37966411

[11] Alharbi R Alsuhaibani K Almarshad A Alyahya A. Depression and anxiety among high school student at qassim region. J Family Med Prim Care. (2019) 8:504–10. doi: 10.4103/jfmpc.jfmpc_383_18 PMC6436297 30984663

[12] Tao Y Tang Q Zou X Wang S Ma Z Liu X. The impact of long-term online learning on internet addiction symptoms among depressed secondary school students: insights from a cross-panel network analysis. Behav Sci (Basel). (2023) 13:1–10. doi: 10.3390/bs13070520 PMC10376411 37503967

[13] Ni F Zheng Y Qian S Shen G Yan W Wu Y. Mental toughness in adolescents: bridging family relationships and depression across personality traits. BMC Psychol. (2024) 12:213. doi: 10.1186/s40359-024-01702-z 38632630 PMC11025235

[14] Zheng M Guo X Chen Z Deng J Hu M. Association between interpersonal relations and anxiety, depression symptoms, and suicidal ideation among middle school students. Front Public Health. (2023) 11:1053341. doi: 10.3389/fpubh.2023.1053341 36866094 PMC9971595

[15] Veloso-Besio C Cuadra-Peralta A Gallardo-Peralta L Cuadra-Fernandez P Quiroz PT Troncoso NV. The prevalence of suicide attempt and suicidal ideation and its relationship with aggression and bullying in Chilean adolescents. Front Psychol. (2023) 14:1133916. doi: 10.3389/fpsyg.2023.1133916 37275702 PMC10234288

[16] Fonseca-Pedrero E Diez-Gomez A de la Barrera U Sebastian-Enesco C Ortuno-Sierra J Montoya-Castilla I. Suicidal behaviour in adolescents: a network analysis. Span J Psychiatry Ment Health. (2024) 17:3–10. doi: 10.1016/j.rpsm.2020.04.007 32493673

[17] Islam MI Yunus FM Kabir E Khanam R. Evaluating risk and protective factors for suicidality and self-harm in Australian adolescents with traditional bullying and cyberbullying victimizations. Am J Health Promot. (2022) 36:73–83. doi: 10.1177/08901171211034105 34308672

[18] Sun R Mendez D Warner KE. The association between cannabis use and subsequent nicotine electronic cigarette use among us adolescents. J Adolesc Health. (2023) 73:133–40. doi: 10.1016/j.jadohealth.2023.02.011 37031094

[19] Azami MS Taremian F. Victimization in traditional and cyberbullying as risk factors for substance use, self-harm and suicide attempts in high school students. Scand J Child Adolesc Psychiatr Psychol. (2020) 8:101–09. doi: 10.21307/sjcapp-2020-010 PMC7863724 33564626

[20] Dadras O Takashi N. Traditional, cyberbullying, and suicidal behaviors in argentinian adolescents: the protective role of school, parental, and peer connectedness. Front Psychiatry. (2024) 15:1351629. doi: 10.3389/fpsyt.2024.1351629 38501081 PMC10944925

[21] Hemono R Gatare E Kayitesi L Hunter LA Kunesh J Packel L. Measuring sexual behavior among in-school youth in Rwanda: a cross-sectional analysis of self-reported timing of first sex and correlates of early sexual debut. Ann Epidemiol. (2023) 83:35–9. doi: 10.1016/j.annepidem.2023.04.005 37060934

[22] Albobali Y Madi MY. Masturbatory guilt leading to severe depression. Cureus. (2021) 13:e13626. doi: 10.7759/cureus.13626 33816025 PMC8011625

[23] Kim H Park KH Park S. Gender differences in sexual behaviors and their relevance to mental health among high school students with sexual experience in South Korea. Int J Environ Res Public Health. (2021) 18:1–9. doi: 10.3390/ijerph182111295 PMC8582716 34769809

[24] Baiden P Mengo C Small E. History of physical teen dating violence and its association with suicidal behaviors among adolescent high school students: results from the 2015 youth risk behavior survey. J Interpers Violence. (2021) 36:NP9526–47. doi: 10.1177/0886260519860087 31271096

[25] Loiselle M Travis F. Improving physical and mental health of college students through consciousness-based education. J Am Coll Health. (2023) 71:2673–78. doi: 10.1080/07448481.2021.1984245 34670104

[26] Park S Park SY Jang SY Oh G Oh IH. The neglected role of physical education participation on suicidal ideation and stress in high school adolescents from South Korea. Int J Environ Res Public Health. (2020) 17:1–11. doi: 10.3390/ijerph17082838 PMC7215916 32326078

[27] Zurita-Ortega F Alonso-Vargas JM Puertas-Molero P Gonzalez-Valero G Ubago-Jimenez JL Melguizo-Ibanez E. Levels of physical activity, family functioning and self-concept in elementary and high school education students: a structural equation model. Children (Basel). (2023) 10:1–10. doi: 10.3390/children10010163 PMC9856633 36670713

[28] Zhang G Feng W Zhao L Zhao X Li T. The association between physical activity, self-efficacy, stress self-management and mental health among adolescents. Sci Rep. (2024) 14:5488. doi: 10.1038/s41598-024-56149-4 38448518 PMC10917799

[29] Livingston JA Chen CH Kwon M Park E. Physical and mental health outcomes associated with adolescent e-cigarette use. J Pediatr Nurs. (2022) 64:1–17. doi: 10.1016/j.pedn.2022.01.006 35121206

[30] Becker TD Arnold MK Ro V Martin L Rice TR. Systematic review of electronic cigarette use (vaping) and mental health comorbidity among adolescents and young adults. Nicotine Tob Res. (2021) 23:415–25. doi: 10.1093/ntr/ntaa171 32905589

[31] Thepthien BO Tinn CS Ofuchi T Kim B. An analysis of e-cigarette and polysubstance use patterns of adolescents in bangkok, Thailand. Tob Induc Dis. (2021) 19:88. doi: 10.18332/tid/142894 34824571 PMC8582419

[32] Jacobs W Idoko E Montgomery L Smith ML Merianos AL. Concurrent e-cigarette and marijuana use and health-risk behaviors among u.s. High school students. Prev Med. (2021) 145:106429. doi: 10.1016/j.ypmed.2021.106429 33476680 PMC8194044

[33] Lau L Conti AA Hemmati Z Baldacchino A. The prospective association between the use of e-cigarettes and other psychoactive substances in young people: a systematic review and meta-analysis. Neurosci Biobehav Rev. (2023) 153:105392. doi: 10.1016/j.neubiorev.2023.105392 37714228

[34] Kinoshita Y Itani O Otsuka Y Matsumoto Y Nakagome S Kaneita Y. A nationwide cohort study of parasomnias among adolescents. J Clin Psychiatry. (2021) 82. doi: 10.4088/JCP.20m13648 34232579

[35] Wang W Du X Guo Y Li W Teopiz KM Shi J. The associations between sleep situations and mental health among chinese adolescents: a longitudinal study. Sleep Med. (2021) 82:71–7. doi: 10.1016/j.sleep.2021.03.009 33901928

[36] Zhang SC Yang R Li DL Wan YH Tao FB Fang J. Association of health literacy and sleep problems with mental health of chinese students in combined junior and senior high school. PloS One. (2019) 14:e0217685. doi: 10.1371/journal.pone.0217685 31173621 PMC6555521

[37] Huang Y Lou H Song Y Cui L Li R Gao G. The association between various dimensions of sleep parameters and mental health: a large cross-sectional study of 13554 chinese students. J Psychosom Res. (2023) 170:111356. doi: 10.1016/j.jpsychores.2023.111356 37178473

[38] Lebni JY Toghroli R Abbas J NeJhaddadgar N Salahshoor MR Mansourian M. A study of internet addiction and its effects on mental health: a study based on Iranian university students. J Educ Health Promot. (2020) 9:205. doi: 10.4103/jehp.jehp_148_20 33062738 PMC7530416

[39] Tomaszek K Muchacka-Cymerman A. Sex differences in the relationship between student school burnout and problematic internet use among adolescents. Int J Environ Res Public Health. (2019) 16:2–10. doi: 10.3390/ijerph16214107 PMC6862502 31653105

[40] Ye XL Zhang W Zhao FF. Depression and internet addiction among adolescents:a meta-analysis. Psychiatry Res. (2023) 326:115311. doi: 10.1016/j.psychres.2023.115311 37348449

